# Editorial: Metabolic Regulation of Drug Resistance and Pathogenicity in Aquatic Pathogens

**DOI:** 10.3389/fmicb.2021.832756

**Published:** 2022-02-03

**Authors:** Bo Peng, Xinhua Chen, Hetron Mweemba Munang'andu

**Affiliations:** ^1^State Key Laboratory of Biocontrol, Guangdong Key Laboratory of Pharmaceutical Functional Genes, School of Life Sciences, Southern Marine Science and Engineering Guangdong Laboratory (Zhuhai), Higher Education Mega Center, Sun Yat-sen University, Guangzhou, China; ^2^Key Laboratory of Marine Biotechnology of Fujian Province, Institute of Oceanology, Fujian Agriculture and Forestry University, Fuzhou, China; ^3^Laboratory for Marine Biology and Biotechnology, Qingdao National Laboratory for Marine Science and Technology, Qingdao, China; ^4^Department of Production Medicine and Science, Section of Experimental Bio-Medicine, Norwegian University of Life Sciences, Ås, Norway

**Keywords:** metabolic regulation, antibiotic resistance, pathogenicity, aquaculture, bacterial pathogen

Aquaculture is an important sector in the food industry able to provide micronutrients to humans, especially in the context of the decline of wild stock of aquatic animals. One big challenge for aquaculture is the infectious diseases that are hard to treat due to the emergence of antibiotic-resistant bacteria (ARB). However, this aspect of ARB has been largely ignored in the past. So that antibiotics are overused to control infectious diseases caused by ARB. Therefore, strategies should be developed to not only manage the antibiotic resistance, but also develop alternative means to control bacterial infection in an antibiotic-independent way, e.g., anti-infective strategy.

Microbial metabolism plays central roles in regulating biological processes including drug resistance and pathogenicity. Bacterial pathogens become resistant to antimicrobials using different mechanisms such as mutations in the cell wall leading to restrictions of drug permeability hindering access to target sites, active efflux of the drug from cells, acquisition of alternative pathways inhibiting drug entry, as well as modification of drug targets (Van Hoek et al., 2011). Metabolic alterations that render microbes to become more pathogenic or refractory to antimicrobials are important mediators of pathogenicity or drug resistance. Therefore, rewiring these metabolic alterations is the key to finding effective strategies for preventing drug resistance and reducing the virulence of pathogens in aquatic organisms. To address these knowledge gaps, this Research Topic aimed at gathering contributions to the Frontiers Research Topic on “*Metabolic Regulation of Drug Resistance and Pathogenicity in Aquatic Pathogens*” for which we were honored to serve as Guest Editors. We gathered a total of 12 articles that present state-of-the-art knowledge on the Research Topic. As outlined below, the articles gathered were broadly grouped into metabolic regulation of (i) drug resistance, and (ii) pathogenicity.

The use of high throughput sequencing (HTS) technologies permits identification of numerous antimicrobial resistance (AMR) genes and pathways that regulate drug resistance. Thus, there exist several platforms and databases for identification of AMR genes as shown by Sakulworakan et al. who analyzed the resistome of *Aeromonas veronii* isolated from diseased tilapia and detected 20 antibiotic resistance genes (ARG)s of which 16 were shared among *A. veronii* populations worldwide. Fu et al. used the data-independent acquisition (DIA) quantitative proteomics method and found a total of 594 differentially expressed genes (DEGs) between the mutant (Δ*yeeY*) and wild-type strain under Furazolidone (FZ) treatment. Using the bacterial drug resistance gene database (CARD), 34 AMR genes were found to be regulated by the YeeY mutant. Several biological pathways such as the secretion system and protein transport were involved in FZ resistance. Liu et al. reviewed the methods used for combating antibiotic tolerance and distinguished antibiotic resistance from tolerance. They showed that exogenous metabolites including amino acids, tricarboxylic acid cycle (TCA cycle) metabolites, and nucleotides effectively activate bacterial metabolism and convent the tolerant cells to sensitive cells.

Gene deletions and knockout systems are the key to elucidating the functional roles of genes that regulate drug metabolism. Fu et al. used gene deletion antibiotics susceptibility assays to show that the YeeY mutant encoded vital genes that regulate FZ resistance in *A. hydrophila*. By comparing protein profiles of *ahslyA* knockout and wild type in *A. hydrophila* strains expressed by the transcriptional regulators (TRs) enoxacin (ENX), Li Z. et al. showed that *ahslyA* deletion upregulated antibiotic resistance proteins of *A. hydrophila* upon ENX stress, pointing to the vital role of *ahslyA* in regulating antibiotic resistance in bacteria. Similarly, Wang et al. showed that deletion of *Hfq* in *A. veroni*i led to increased trimethoprim resistance accompanied by downregulation of the efflux pump related genes acrA and acrB while complementation of acrA and acrB in Δhfq reversed the resistance. This study demonstrated that Hfq mediated trimethoprim resistance by elevating the efflux pump expression. Altogether, these studies underscore the importance of mutational changes in regulating drug metabolism.

Antibiotic resistance or tolerance is strongly correlated with bacterial metabolism, which is an attractive spot for developing intervening approaches for control antibiotic resistance. Ortiz-Severín et al. showed that nutrient scarcity can lead to antibiotic resistance. They showed that all *Piscirickettsia salmonis* strains grown in nutrient-limited media were resistant to ampicillin, erythromycin, penicillin G, streptomycin, spectinomycin, polymyxin B, ceftazidime, and trimethoprim in a nutrient deficiency dependent manner. Ampicillin resistance was linked to decrease in bacterial metabolism that included the TCA cycle, pentose-phosphate pathway, energy production, and nucleotide metabolism. Contrariwise, some chemical formulations and metabolites have been shown to enhance bacteria susceptibility to drug treatment. For example, Gao et al. showed that *A. hydrophila, Vibrio harveyi, V. fluvialis, V. alginolyticus, E. tarda*, and *Streptococcus iniae* can be rapidly killed during their stationary growth phase after immersion in gentamicin- or neomycin-containing ion-free solutions. They noted that hypoionic shock enhanced bacterial uptake of gentamicin in an ATP-dependent manner thereby enhancing the killing effect of *A. hydrophila* in infected zebrafish (*Danio rerio*). In another study, Srinivasan et al. showed that naringin (NA) can be used as an anti-quorum sensing (QS) compound resulting in reduced biofilm formation in *A. hydrophila*. In zebrafish infected with *A. hydrophila* the recovery rate increased upon NA treatment. Altogether, these studies show that factors that influence bacteria growth such as nutrient scarcity, metabolites like NA as well as compounds like hypo-ionic solutions have a significant influence on bacteria susceptibility or resistance to antimicrobials.

Discovery of novel genes that regulate pathogenicity is essential for understanding the cellular mechanisms of disease establishment. Li D. Y. et al. identified a novel T6SS effector, named EvpQ, encoded by mobile genetic elements in the *E. piscicida* genome. Sequence analysis reveals that EvpQ shares a conserved domain of C70 family cysteine protease with the T3SS effector AvrRpt2 of phytopathogenic *Erwinia amylovora*. Discovery of the EvpQ gene in the T6SS effector is bound to enhance our understanding of the pathogenicity of T6SS in edwardsiellosis.

Elucidating gene functions that regulate bacteria pathogenicity is sometimes better explained using gene knockouts or mutant strains. After characterizing the type II TA system based on the YefM-YoeB gene of *E. piscicida* where YoeB is the toxin shown to arrest bacterial growth restored by the adding the antitoxin YefM, Ma et al. constructed *yoeB* and *yefM-yoeB* in-frame mutant strains of which *yefM-yoeB* was shown to reduce resistance against oxidative stress and antibiotics while its deletion enhanced bacterial high temperature tolerance, biofilm formation, and host serum resistance. In addition, *yefM-yoeB* was shown to enhance bacterial host cells adhesion, dissemination, and virulence in fish tissues. In another study, Hu et al. investigated the effect of the *tonB* gene on the virulence of *Pseudomonas plecoglossicida* by knocking down the *tonB* with RNAi. The differences between the wild-type and *tonB*-RNAi strains showed that *tonB* regulates virulence through motility, chemotaxis, adhesion, and biofilm formation in *P. plecoglossicida* infections. These studies demonstrated the use of gene knockdown and other mutational changes to underpin the functional roles of genes regulating pathogenicity.

Novel platforms for microbial gene analysis are highly needed for elucidating networks that regulate microbial virulence and drug resistance. Xie et al. developed an online network platform called AbviresDB, integrating co-functional multiple sources of data from *Acinetobacter baumannii*. They used the k-shell decomposition approach to analyze the co-functional network and showed that genes involved in basic cellular physiological function such as drug resistance genes had high k-shell values while non-essential genes like virulence genes had lower k-shell values. They showed that ABviresDB platform can be used for visualization of each gene in the network having the potential for drug resistance and pathogenesis research.

Altogether, this Research Topic outlines metabolic regulations that can be exploited to overcome drug resistance and pathogenicity of pathogens infecting aquatic organisms. We are confident that the information availed in the articles will advance our understanding of the metabolic regulation of drug resistance and pathogenicity. We also envisage that data presented in these articles will contribute to developing preventive measures against drug resistance and to reducing the prevalence of infectious diseases of aquatic organisms.

## Author Contributions

All authors listed have made a substantial, direct, and intellectual contribution to the work and approved it for publication.

## Funding

This work was sponsored by grants from NSFC project (Nos. 32061133007 and U1905204), China Agriculture Research System (CARS-47), Project supported by Innovation Group Project of Southern Marine Science and Engineering Guangdong Laboratory Zhuhai (No. 311020006), and Research Council of Norway RCN (No. 320692).

## Conflict of Interest

The authors declare that the research was conducted in the absence of any commercial or financial relationships that could be construed as a potential conflict of interest.

## Publisher's Note

All claims expressed in this article are solely those of the authors and do not necessarily represent those of their affiliated organizations, or those of the publisher, the editors and the reviewers. Any product that may be evaluated in this article, or claim that may be made by its manufacturer, is not guaranteed or endorsed by the publisher.
